# A Molecular Clue to PTSD

**DOI:** 10.1371/journal.pbio.1002283

**Published:** 2015-10-27

**Authors:** Richard Robinson

**Affiliations:** Freelance Science Writer, Sherborn, Massachusetts, United States of America

## Abstract

Work on rats and humans reveals a role for reduction in the levels of the stress-regulated protein kinase SGK1 in the development of post-traumatic stress disorder. Read the Research Article.

Post-traumatic stress disorder (PTSD) is one of the most common psychic injuries in trauma victims, including soldiers after combat. Untreated, the fear, intrusive thoughts, hyperarousal, and emotional numbing it causes can last a lifetime. While progress has been made in understanding the dysregulation of brain circuits in PTSD, developing treatments has nonetheless remained challenging because of lack of insight into the molecular and cellular mechanisms underlying these changes. In a new study in this issue of *PLOS Biology*, Pawel Licznerski, Ronald Duman, and colleagues begin to reveal those mechanisms, showing that the function of a stress-regulated protein is reduced in the prefrontal cortex in people with PTSD and that reducing function of the protein in rats induces PTSD-like behavior.

The authors began their work with a whole-genome array screen for up- or down-regulated genes in the postmortem brains of six subjects with PTSD and an equal number of controls. (The small number of cases, which represent the totality of samples in the first PTSD brain bank, speaks to an unmet need in the field.) One of the most dysregulated genes, and one of the few to survive stringent statistical analysis, was serum and glucocorticoid regulated kinase 1 (SGK1), whose expression in the prefrontal cortex of PTSD subjects was reduced by over 80% compared to controls. Both the gene and its location appeared significant: SGK1 is controlled in part by stress hormones, and reduced activity of the prefrontal cortex has been shown to be a major feature in the brains of people affected by PTSD.

To understand the behavioral implications of reduced SGK1, the authors studied rats under stress. Rats who receive a shock to the foot but are prevented from escaping develop a helplessness that later leads them to remain in place and receive a shock, even when they could escape. The degree of helplessness (a form of which is seen in PTSD) varies among individual rats, and the authors found that those least likely to flee when they could had the lowest levels of SGK1 activity in their prefrontal cortices. That same helplessness could be induced by delivering a viral vector to the brain carrying a mutant gene that inhibited SGK1 function. Conversely, overexpression of normal SGK1 led to an increase in escape activity.

Reducing SGK1 also reduced the rats’ interest in sugar water, a manifestation of anhedonia, one characteristic of PTSD. During a fear conditioning and extinction test, rats with down-regulated SGK1 froze more often in response to the original fear stimulus, as long as the stimulus was presented in its original context, suggesting an enhancement of context-driven fear response. At the cellular level, reduction of SGK1 activity decreased the density of dendritic spines on neurons in the prefrontal cortex, with a consequent reduction in synaptic activity. The reduction in prefrontal activity seen in PTSD patients, and replicated in part here in rats, is believed to reduce inhibitory control over the amygdala, involved in the brain’s fear circuit (Fig 1).

**Fig 1:**
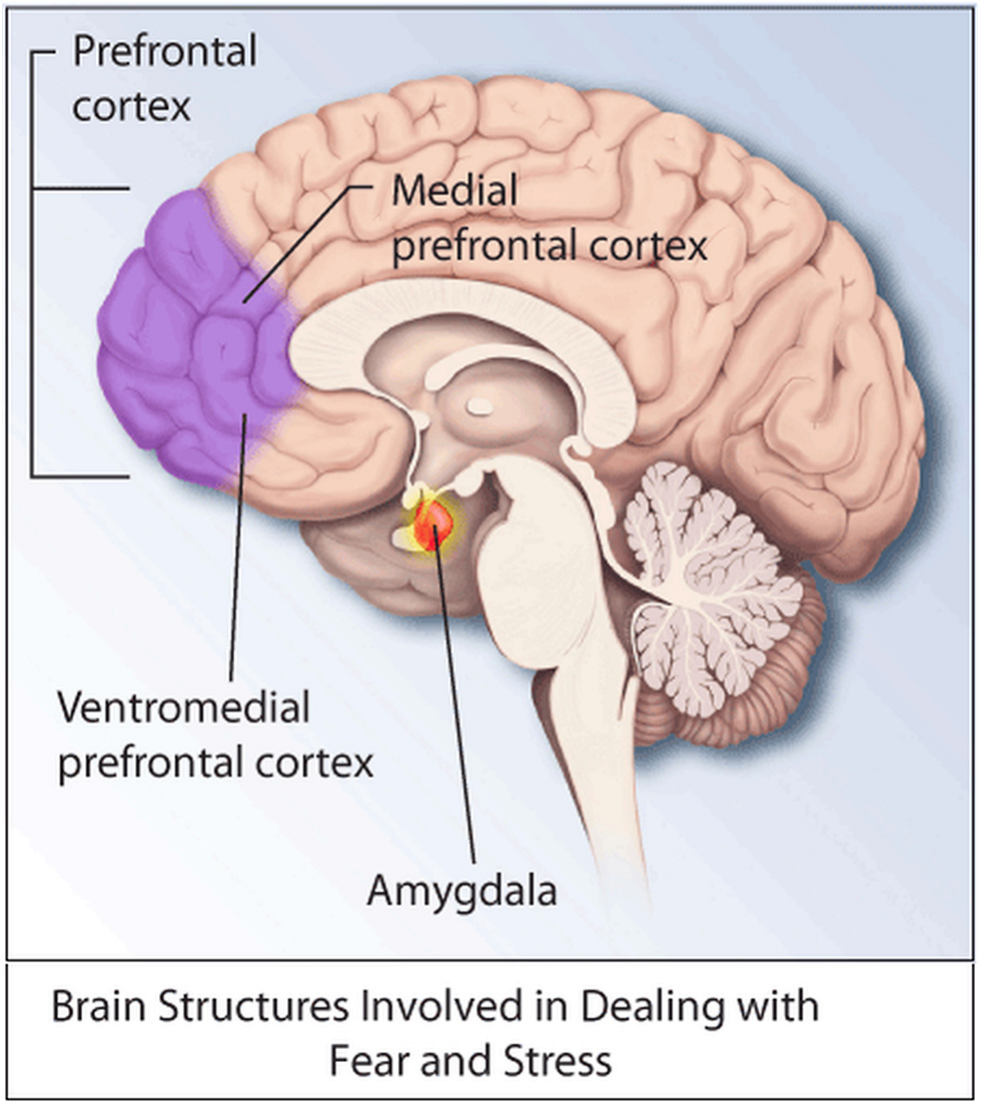
Stress-induced reduction of SGK1 activity was found to reduce synaptic activity in the prefrontal cortex, which is thought to decrease inhibitory control over the amygdala, a major component of the brain’s fear circuit. *Image credit*: *NIH Medical Arts*.

There is much left to be understood about the molecular and cellular underpinnings of PTSD, but if these results can be replicated in a larger sample, they may provide a major insight into how the brain responds to traumatic stress and how it might be healed. Drugs that increase SGK1 activity, or mimic it, might be therapeutic, and further investigation of the changes induced by lowered SGK1 activity are sure to reveal other and perhaps more easily manipulated drug targets.

## Abbreviations

PTSD: post-traumatic stress disorder
SGK1: serum and glucocorticoid regulated kinase 1
